# Hepatocyte growth factor (HGF) and stem cell factor (SCF) maintained the stemness of human bone marrow mesenchymal stem cells (hBMSCs) during long-term expansion by preserving mitochondrial function via the PI3K/AKT, ERK1/2, and STAT3 signaling pathways

**DOI:** 10.1186/s13287-020-01830-4

**Published:** 2020-07-31

**Authors:** Zeyuan Cao, Yunyi Xie, Le Yu, Yi Li, Yan Wang

**Affiliations:** Hospital of Stomatology, Guanghua School of Stomatology, Guangdong Provincial Key Laboratory of Stomatology, Sun Yat-sen University, 56 Lingyuanxi Road, Guangzhou, 510055 China

**Keywords:** Stem cells from human exfoliated deciduous teeth, Hepatocyte growth factor, Stem cell factor, Stemness, Senescence, Osteogenic differentiation, Mitochondrial function

## Abstract

**Background:**

Mesenchymal stem cells (MSCs) have a limited self-renewal ability, impaired multi-differentiation potential, and undetermined cell senescence during in vitro series expansion. To address this concern, we investigated the effects of the microenvironment provided by stem cells from human exfoliated deciduous teeth (SHED) in maintaining the stemness of human bone marrow mesenchymal stem cells (hBMSCs) and identified the key factors and possible mechanisms responsible for maintaining the stemness of MSCs during long-term expansion in vitro.

**Methods:**

The passage 3 (P3) to passage 8 (P8) hBMSCs were cultured in the conditioned medium from SHED (SHED-CM). The percentage of senescent cells was evaluated by β-galactosidase staining. In addition, the osteogenic differentiation potential was analyzed by reverse transcription quantitative PCR (RT-qPCR), Western blot, alizarin red, and alkaline phosphatase (ALP) staining. Furthermore, RT-qPCR results identified hepatocyte growth factor (HGF) and stem cell factor (SCF) as key factors. Thus, the effects of HGF and SCF on mitochondrial function were assessed by measuring the ROS and mitochondrial membrane potential levels. Finally, selected mitochondrial-related proteins associated with the PI3K/AKT, ERK1/2, and STAT3 signaling pathways were investigated to determine the effects of HGF and SCF in preserving the mitochondrial function of hBMSCs during long-term expansion.

**Results:**

SHED-CM had significantly enhanced the cell proliferation, reduced the senescent cells, and maintained the osteogenesis and pro-angiogenic capacity in P8 hBMSCs during long-term expansion. In addition, hBMSCs treated with 100 ng/ml HGF and 10 ng/ml SCF had reduced ROS levels and preserved mitochondrial membrane potential compared with P8 hBMSCs during long-term expansion. Furthermore, HGF and SCF upregulated the expression of mitochondrial-related proteins associated with the PI3K/AKT, ERK1/2, and STAT3 signaling pathways, possibly contributing to the maintenance of hBMSCs stemness by preserving mitochondrial function.

**Conclusion:**

Both HGF and SCF are key factors in maintaining the stemness of hBMSCs by preserving mitochondrial function through the expression of proteins associated with the PI3K/AKT, ERK1/2, and STAT3 signaling pathways. This study provides new insights into the anti-senescence capability of HGF and SCF, as well as new evidence for their potential application in optimizing the long-term culture of MSCs.

## Introduction

In recent years, several studies have focused on the various therapeutic applications of mesenchymal stem cells (MSCs), including cell transplantation for cartilage and bone repair [1, 2] and neuronal regeneration [3, 4], due to their ability to self-renew and differentiate into a variety of cell types. Furthermore, due to their low immunogenicity and ability to secrete immune factors or cytokines [5, 6], MSCs have been studied and utilized in clinical trials for some immune-related diseases, such as chronic graft versus host diseases (cGVHD) [7] and Crohn’s disease [8].

The number of MSCs available is limited by the donor tissues or organs. Hence, the expansion of MSCs in vitro is necessary. However, their unpredictable differentiation, senescence, and loss of stemness during in vitro culture reduce their effectiveness and create unknown risks [9, 10]. In particular, the human bone marrow mesenchymal stem cells (hBMSCs) are difficult to expand in vitro and prone to cell aging, possibly due to the difficulty of simulating their growth environment in vivo [11, 12]. Stem cells reside in a specific microenvironment called the stem cell niche. This microenvironment consists of the extracellular matrix (ECM) and neighboring cells, such as endothelial, fibroblast, and other progenitor cells [13]. In addition, the stem cells essentially contribute a large number of bioactive molecules, such as cytokines, chemokines, angiogenic factors, and growth factors to the niche. The stem cell niche, which is maintained by a continuous communication between the stem cells and the surrounding cells through cell signaling or the paracrine effects, determines stem cell fate to maintain a balance of self-renewal and differentiation and influences cell senescence or apoptosis [14–16].

It is necessary to devise a way to maintain the stemness of MSCs during long-term expansion and simultaneously produce MSCs in sufficient quantity for therapeutic purposes. Previous studies have addressed this problem by mimicking the in vivo stem cell niche to optimize cell culture conditions and prepare a sufficient quantity of qualified pluripotent stem cells. Currently, there are several methods to maintain the stemness of MSCs. For example, many biomaterials have been invented to promote the proliferation and self-renewal ability of MSCs. Some of these biomaterials contain laminin, collagen, and fibronectin, which are the primary composition of the ECM [17, 18]. On the other hand, 3D cell culture models provide additional dimensions for cell adhesion and mimic in vivo cell morphology and molecular control [19]. In addition, co-culture systems with other cell types, such as endothelial progenitor cells [20] are considered into cell culture gradually for mimic an in vivo cellular niche, which have gotten some notable effect.

Several studies have observed that stem cells from human exfoliated deciduous teeth (SHED) have significantly better and more sustained ability to proliferate and resist senescence than hBMSCs [21, 22]. In addition, as the factors secreted by MSCs have a crucial role in maintaining their stemness, we speculate that this may also be true for SHED. Therefore, we hypothesized that hBMSCs cultured using SHED-CM in vitro may afford them with enhanced proliferative and self-renewal properties during long-term expansion. The secretions of SHED were also investigated further to identify the key factors and possible mechanisms associated with the stemness of hBMSCs and potentially devise a new method to optimize the long-term expansion of MSCs in vitro.

## Methods and materials

### Cell isolation and culture

SHED were isolated and collected from non-caries exfoliated human deciduous teeth (4–10 years old; 6 males and 6 females, without oral or systematic diseases) after informed consent and approved by the Ethics Committee of Hospital of Stomatology in Sun Yat-sen University. Briefly, the pulps from deciduous teeth were minced and digested with 3 mg/ml collagenase type I and 4 mg/ml dispase (Gibco-BRL, USA) and cultured using DMEM (Gibco-BRL, USA) that contained 10% FBS, 100 units/ml streptomycin (HyClone, USA), and 100 units/ml penicillin (HyClone, USA). hBMSCs at passage 2 (P2) were bought from Cyagen Biosciences (China), from healthy adults (18–45 years old), and cultured using DMEM that contained 10% FBS, 100 units/ml streptomycin, and 100 units/ml penicillin. Human umbilical vein endothelial cells (HUVECs) were bought from the China Center for Type Culture Collection (CCTCC, China) and cultured using endothelial cell medium (ECM, ScienCell, USA) that contained 5% FBS, 100 units/ml streptomycin, and 100 units/ml penicillin. Hepatocyte growth factor (HGF), stem cell factor (SCF), and insulin-like growth factor 2 (IGF2) (all from PeproTech, USA) were supplemented into DMEM during the long-term expansion of hBMSCs from passage 3 (P3) to passage 8 (P8). LY294002 (Sigma-Aldrich), U0126 (MedChemExpress), and Stattic (MedChemExpress) were separately added to the culture medium to block PI3K/AKT, ERK1/2, and STAT3 signaling pathways.

### Supernatant collection

SHED (P3) or hBMSCs (P3) were seeded into a 10-cm dish (4 × 10^5^ cells per dish) and cultured in DMEM that contained 10% FBS for 3 days. The supernatants of SHED or hBMSCs were collected (centrifuged at 800 g for 5 min to remove cell debris) and preserved at − 80 °C. For supplying enough nutrition to cells, the supernatants were mixed with fresh DMEM that contained 10% FBS in a 1:1 ratio when used, named as conditioned medium from SHED (SHED-CM) or conditioned medium from hBMSCs (hBMSCs-CM). To explore the effect of conditioned mediums on stemness and senescence of hBMSCs during long-term expansion, hBMSCs were cultured in DMEM, SHED-CM, or hBMSCs-CM from P3 to P8, respectively.

### Colony-forming unit (CFU) assay

To detect the effect of conditioned mediums on the self-renewal ability of hBMSCs (P3), 1000 cells were seeded into a 10-cm dish in each group and cultured in DMEM that contained 10% FBS, SHED-CM, or hBMSCs-CM for 14 days. hBMSCs were washed with PBS, fixed with 4% paraformaldehyde (PFA), and stained with 0.1% (w/v) crystal violet (Sigma, USA).

### Cell cycle analysis

hBMSCs in each group (P3, P8, P8-SHED-CM and P8-hBMSCs-CM) were seeded into 6-well culture plates (5 × 10^4^ cells per well) and collected after 3 days. Cells were washed with cold PBS and fixed in 70% ethanol overnight at 4 °C. After being washed with cold PBS, cells were incubated with 50 μg/ml propidium iodide (PI), 100 μg/ml ribonuclease A, and 0.1% TritonX-100 in PBS at 4 °C for 30 min and detected by flow cytometry (Beckman Coulter, Germany). Data were analyzed by Flowjo software. The Dean-Jett-Fox model was used to calculate the cell cycle phases.

### β-Galactosidase staining

hBMSCs in each group (P3, P8, P8-SHED-CM and P8-hBMSCs-CM) were seeded into 6-well culture plates (5 × 10^4^ cells per well) and cultured for 3 days. Senescent cells were stained by a β-galactosidase (β-gal) staining kit (Beyotime, China) according to the manufacturer’s instructions. The β-gal-positive cells were scanned by microscopy, and 6 fields were taken to calculate the proportion of senescent cells in each group.

### Osteogenic induction

For RT-qPCR and Western blot assay, hBMSCs in each group were cultured into 6-well plates (5 × 10^4^ cells per well) for 3 days, followed by induced with osteogenic differentiation medium (DMEM that contained 10% FBS, 10 mM β-glycerophosphate, 10 nM dexamethasone, and 50 μg/mL ascorbic acid) for 7 days. For alizarin red and alkaline phosphatase (ALP) staining, hBMSCs in each group were cultured into 24-well plates (1 × 10^4^ cells per well) for 3 days, followed by induced with osteogenic differentiation medium for 14 days. P3 hBMSCs without osteogenic induction were named as “P3”, with induction named as “P3+”. Similarly, other groups were named by the same way (as P8, P8+, P8-SHED-CM+, P8-hBMSCs-CM+).

### Total RNA extraction and reverse transcription quantitative PCR (RT-qPCR)

Total RNA of cells was isolated with RNA isolation kit (Ultrapure RNA Kit, CW Biotech, China) and transcribed into cDNA using Reverse Transcriptase M-MLV kit (TaKaRa, China). RT-qPCR assay was performed according to the manufacturer’s instructions of SYBR Green PCR Master Mix kit (Roche, Switzerland). GAPDH was used as the internal control. The cycling conditions were all as follows: incubation at 95 °C for 10 min, 40 cycles of denaturation at 95 °C for 15 s, annealing at 60 °C for 20s, and extension at 72 °C for 20s. The primer sequences for each gene are listed in Table 1. The expression level of targeted gene was analyzed by 2^−ΔΔCt^ method.

**Table 1:** Primer sequences used in reverse transcription quantitative PCR (RT-qPCR)

Gene	Sequence
p16	Forward: 5′-CCCCTTGCCTGGAAAGATAC-3′
	Reverse: 5′-AGCCCCTCCTCTTTCTTCCT-3′
p21	Forward: 5′-AGCAGCGGAACAAGGAGT-3′
	Reverse: 5′-TTACAGTCTAGGTGGAGAAACG-3′
Nanog	Forward: 5′-AAGGCCTCAGCACCTACCTA-3′
	Reverse: 5′-TGCACCAGGTCTGAGTGTTC-3′
OCT4	Forward: 5′-TGGATGTCAGGGCTCTTTGTC-3′
	Reverse: 5′-ACCTTCCCAAATAGAACCCCC-3′
Runx2	Forward: 5′-TGGTTACTGTCATGGCGGGTA-3′
	Reverse: 5′-TCTCAGATCGTTGAACCTTGCTA-3′
BSP	Forward: 5′-GAACCACTTCCCCACCTTTTG-3′
	Reverse: 5′-ATTCTGACCATCATAGCCATCG-3′
ALP	Forward: 5′-TTCAAACCGAGATACAAGCACT-3′
	Reverse: 5′-GGGCCAGACCAAAGATAGAG-3′
GAPDH	Forward: 5′-GGAGCGAGATCCCTCCAAAAT-3′
	Reverse: 5′-GGCTGTTGTCATACTTCTCATGG-3′

### Western blot analysis

Cells were harvested and lysed in RIPA (50 mM Tris-HCl pH 7.4, 150 mM NaCl, 1%TritonX-100, 0.5% sodium deoxycholate, 0.1% SDS, and protease inhibitor cocktail) on ice for 30 min. Total protein concentrations were measured by a BCA protein assay kit (Pierce, Thermo Scientific). Western blot assay was performed using protocols described below. Briefly, 40 μg total protein was separated by 10% sodium dodecyl sulfate polyacrylamide gel electrophoresis (SDS-PAGE), transferred onto nitrocellulose (NC) membranes, blocked by 5% (w/v) nonfat milk for 1 h at room temperature and then incubated with primary antibodies specific for Runx2 (ABclonal, 1:500), BSP (Boster, 1:200), Mitofusin 1 (Mfn1, Boster, 1:200), Mitofusin 2 (Mfn2, Boster, 1:200), Catalase (Cell Signaling Technology, 1:1000), SOD2 (Cell Signaling Technology, 1:1000), PI3K (Boster, 1:200), phospho-AKT (p-AKT, Cell Signaling Technology, 1:1000), AKT (Cell Signaling Technology, 1:1000), phospho-ERK1/2 (p-ERK1/2, Cell Signaling Technology, 1:2000), Erk1/2 (Cell Signaling Technology, 1:1000), phospho-STAT3 (p-STAT3, Cell Signaling Technology, 1:2000), STAT3 (ABclonal, 1:2000), and β-actin (Sigma 1:5000) overnight at 4 °C. After being incubated with secondary antibodies for 1 h at room temperature, the membranes were scanned by an Odyssey two-color infrared laser imaging system (LI-COR Biosciences, USA). Relative protein expression levels were analyzed by ImageJ software and normalized to β-actin.

### Alizarin red and ALP staining

For alizarin red staining (ARS), hBMSCs in each group (P3, P3+, P8, P8+, P8-SHED-CM+, and P8-hBMSCs-CM+) were fixed with 4% PFA and stained with 1% (w/v) Alizarin Red. The nodules were scanned by microscopy and dissolved by 10% (w/v) cetylpyridinium chloride (Sigma, USA). OD values were measured at 562 nm. ALP staining was performed by the BCIP/NBT Alkaline Phosphatase Color Development Kit (Beyotime Biotech, China), according to the manufacturer’s instructions.

### Cell proliferation detection

The effect of HGF, SCF, and IGF2 on the proliferation ability of hBMSCs was detected using CFSE assay. hBMSCs (P3) were labeled with 1 μM of CFSE (Invitrogen, USA) before culture; then, cells were seeded into 6-well plates (5 × 10^4^ cells per well) and cultured in DMEM supplemented with different factors at different concentrations for 3 days. The proliferative cell rate was detected by flow cytometry (CytoFLEX, Beckman Coulter). Data were analyzed by Flowjo software.

### Assessment of ROS production

hBMSCs in each group (P3, P8, P8-SHED-CM, P8-HGF 100, P8-SCF 10 and P8-H+S) were seeded into 6-well culture plates (5 × 10^4^ cells per well) and cultured for 3 days. Intracellular ROS was detected by fluorescence probe DCFH-DA (Sigma, USA) according to the manufacturer’s protocol. Briefly, cells were incubated with 10 μM DCFH-DA in dark at 37 °C for 20 min, then washed by PBS and observed under fluorescence microscope (Cael Zeiss, Oberkochen, Germany). As a positive control, hBMSCs in passage 3 (P3) were incubated with 1 mM H_2_O_2_ for 1 h prior to the detection [23, 24]. As a negative control, DCFH-DA probe was replaced by an equal-volume of DMSO. To obtain quantitative result, the fluorescence intensity was detected by flow cytometry (CytoFLEX, Beckman Coulter). Data were analyzed with CytExpert Software (Beckman Coulter).

### Mitochondrial membrane potential assay

hBMSCs in each group (P3, P8, P8-SHED-CM, P8-HGF 100, P8-SCF 10 and P8-H+S) were seeded into 6-well culture plates (5 × 10^4^ cells per well) and cultured for 3 days. Mitochondrial membrane potential was detected by a JC-1 fluorescent probe assay kit (Beyotime Biotech, China) according to the manufacturer’s protocol. Cell fluorescence was observed under fluorescence microscope (Cael Zeiss, Oberkochen, Germany). To obtain quantitative results, the fluorescence intensity was detected by flow cytometry (CytoFLEX, Beckman Coulter). Data were analyzed with CytExpert Software (Beckman Coulter).

### Statistical analysis

All data were performed in biological triplicates and results were expressed as mean ± SD. Statistical analysis was conducted using SPSS20.0 software package (SPSS Inc., Chicago, IL, USA). One-way analysis of variance (ANOVA) was used and post hoc Bonferroni test was performed for multiple comparisons. *P* values < 0.05 were considered statistically significant.

## Results

### hBMSCs cultured in SHED-CM had enhanced cell proliferation

**Fig. 1:**
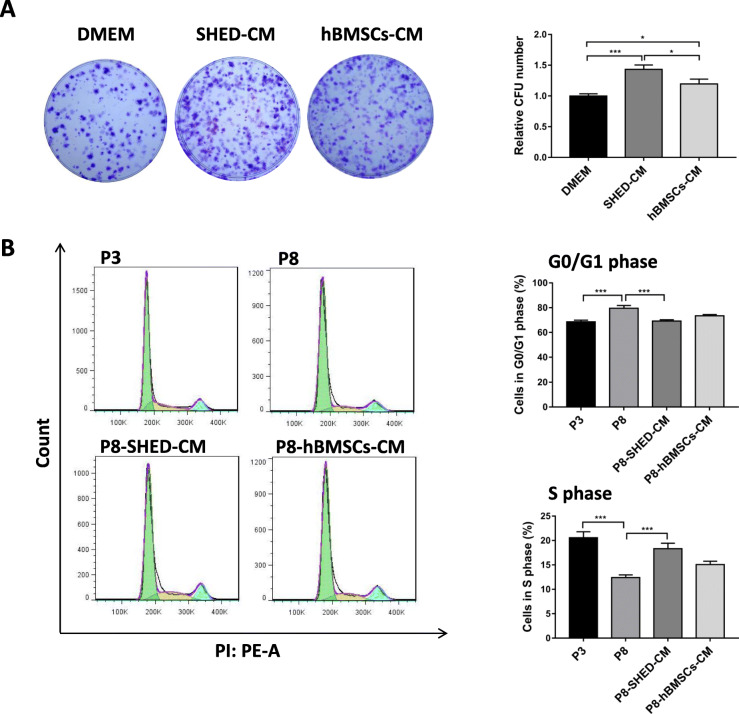
hBMSCs cultured in SHED-CM had enhanced cell proliferation. **a** Representative images of hBMSCs cultured in DMEM, SHED-CM. and hBMSCs-CM, and quantitative analysis of relative CFU number. **b** Cell cycle analysis of passage 3 (P3) and passage 8 (P8) hBMSCs cultured in DMEM, SHED-CM (P8-SHED-CM) and hBMSCs-CM (P8-hBMSCs-CM) after long-term expansion. Percentage (%) of hBMSCs undergoing the G0/G1 and S phases. *P < 0.05, ***P < 0.001

CFU assay was performed to examine the effect of SHED-CM on the self-renewal ability of hBMSCs. Results showed that hBMSCs cultured in SHED-CM had the highest colony number compared with hBMSCs cultured in DMEM and hBMSCs-CM, indicating that SHED-CM significantly enhanced the self-renewal of hBMSCs (Fig. 1a). The cell proliferation after long-term expansion from passage 3 (P3) to passage 8 (P8) in different conditioned mediums was detected by cell cycle assay. Results showed that about 80% hBMSCs had cell cycle arrest in G0/G1 phase at P8, and the S phase population significantly decreased at P8 (12.4%) compared with P3 (20.5%) hBMSCs. SHED-CM treatment decreased the G0/G1 phase population to approximately 70% and induced the hBMSCs to undergo S phase (18.3%) (Fig. 1b). These results demonstrated that SHED-CM can improve the proliferative and self-renewal abilities of hBMSCs during long-term expansion.

### hBMSCs cultured in SHED-CM had less senescence and maintained stemness during long-term expansion

**Fig. 2:**
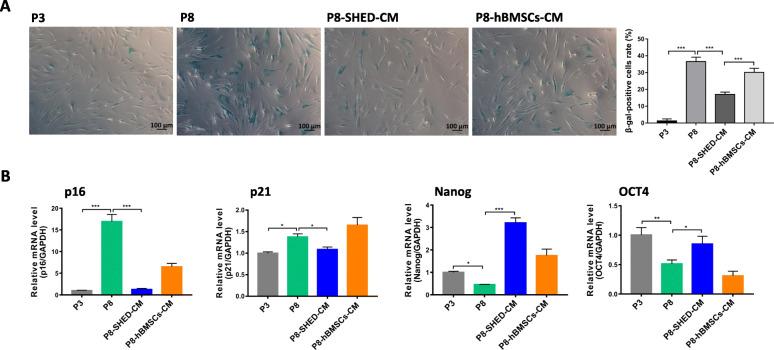
hBMSCs cultured in SHED-CM had less senescence and maintained stemness during long-term expansion. **a** Representative images of β-gal stained passage 3 (P3) and passage 8 (P8) hBMSCs cultured in DMEM, SHED-CM (P8-SHED-CM), and hBMSCs-CM (P8-hBMSCs-CM), and corresponding rate (%) of β-gal-positive cells per group. **b** Relative mRNA expression levels of senescence (*p16* and *p21*) and stemness (*Nanog* and *OCT4*) markers per group. *P < 0.05, **P < 0.01, ***P < 0.001

After confirming that SHED-CM treatment can promote the proliferation and self-renewal of hBMSCs, we also determined the effects of the treatment in regulating the stemness and senescence of hBMSCs during long-term expansion. β-Galactosidase staining assay revealed that hBMSCs in P8-SHED-CM group had fewer β-gal-positive cells (17%) than that in P8 hBMSCs (36.5%) and P8-hBMSCs-CM (30.1%) groups (Fig. 2a). The mRNA expression levels of senescence (*p16* and *p21*) and stemness (*Nanog* and *OCT4*) markers were analyzed using RT-qPCR. P8 hBMSCs had higher *p16* and *p21* expression levels and lower *Nanog* and *OCT4* expression levels than P3 hBMSCs. The *p16* and *p21* expressions were significantly downregulated in P8-SHED-CM hBMSCs, while *Nanog* and *OCT4* were significantly upregulated compared with P8 hBMSCs (Fig. 2b). These results indicated that SHED-CM can potentially delay cell senescence and maintain the stemness of hBMSCs during long-term expansion.

### hBMSCs cultured in SHED-CM had enhanced osteogenic differentiation potential during long-term expansion

**Fig. 3:**
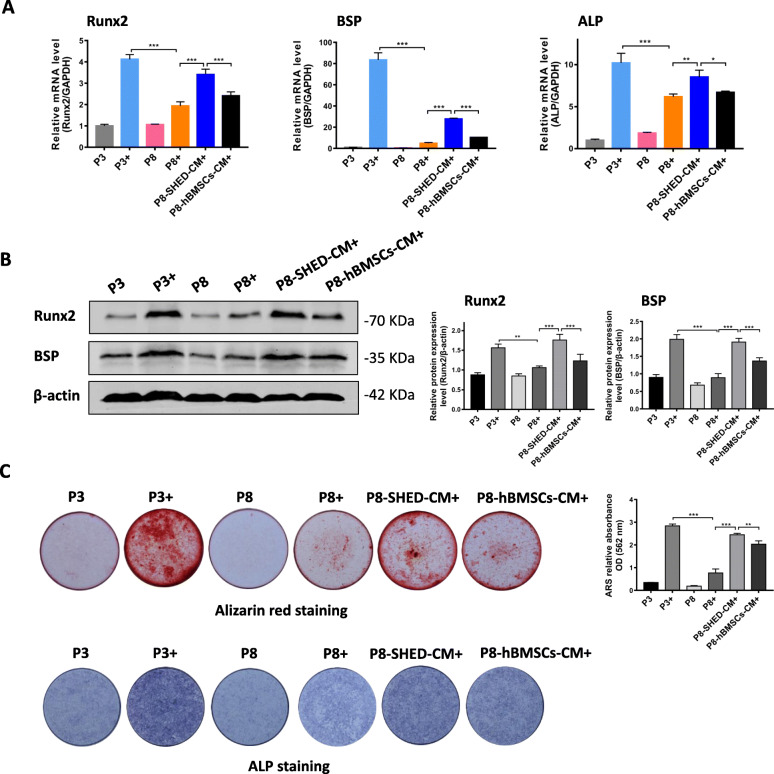
hBMSCs cultured in SHED-CM had enhanced osteogenic differentiation potential during long-term expansion. Results of the analysis on the expression of osteogenic-related proteins in passage 3 (P3) and passage 8 (P8) hBMSCs cultured in DMEM, SHED-CM (P8-SHED-CM), and hBMSCs-CM (P8-hBMSCs-CM). **a** Relative mRNA expression levels of Runx2, BSP, and ALP per treatment group after osteogenic induction for 7 days. **b** Western blot results and relative expression levels of Runx2 and BSP proteins per treatment group after osteogenic induction for 7 days. **c** Alizarin Red staining (ARS) after osteogenic induction for 14 days and the corresponding ARS relative absorbance OD of hBMSCs at 562 nm. ALP staining of hBMSCs after osteogenic induction for 14 days. Plus sign indicates with osteogenic induction. *P < 0.05, **P < 0.01, ***P < 0.001

After osteogenic induction for 7 days, the mRNA expression levels of the osteogenic markers (*Runx2*, *BSP*, and *ALP*) significantly increased in the P3+ group and significantly decreased in the P8+ group. Furthermore, the *Runx2*, *BSP*, and *ALP* mRNA expression levels in the P8-SHED-CM+ group was significantly higher than the P8+ group (Fig. 3a). Western blot analysis revealed significantly decreased expression levels of the osteogenic-related proteins, Runx2, and BSP in P8+ group. Both proteins were highly expressed in the P8-SHED-CM+ group compared with the P8+ group (Fig. 3b). Alizarin red staining showed that there were more calcium deposits in the P8-SHED-CM+ group than the P8+ and P8-hBMSCs-CM+ groups. Correspondingly, results of the alkaline phosphatase (ALP) staining showed the depth of color among the different groups (Fig. 3c). Collectively, these results suggest that SHED-CM provided a better environment for the survival of hBMSCs, resulting in the retention of osteogenic differentiation potential during long-term expansion in vitro.

### Identification of key factors and determination of effective concentrations

To identify the key factors in SHED supernatant that play a role in preventing senescence and enhancing the osteogenic potential of hBMSCs, we selected previously studied factors secreted by SHED [25, 26] or factors regulating the proliferation, stemness, or differentiation of MSCs and compared their expression levels among several oral-derived MSCs and hBMSCs in P3 and P8 using RT-qPCR (Supplemental Data 2). We hypothesized that factors with high expression in SHED and low expression in hBMSCs during series expansion were potential key factors. RT-qPCR results revealed three potential key factors specifically, HGF, SCF, and IGF2. We hypothesized that these factors in SHED-CM may play a role in the delayed senescence and enhanced osteogenic differentiation of hBMSCs.

**Fig. 4:**
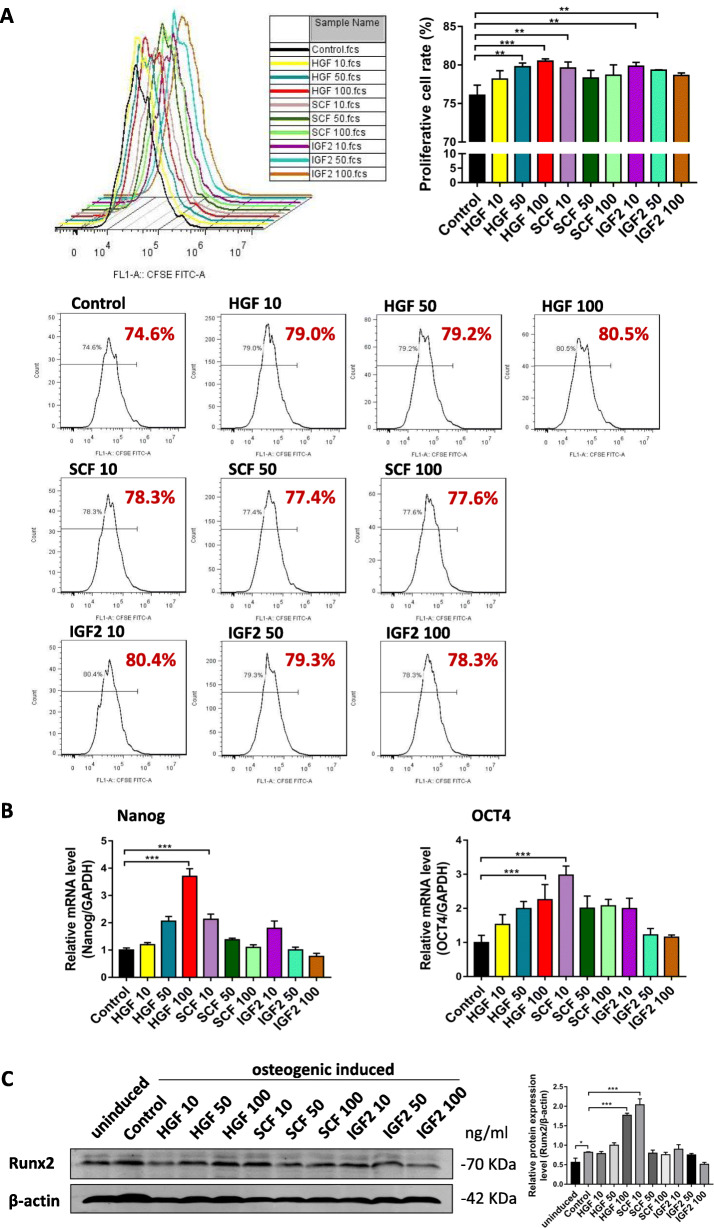
Identification of key factors and determination of effective concentrations. **a** Flow cytometry results and individual graphs of CFSE assay, and corresponding proliferative cell rate (%) of hBMSCs treated with 10 ng/ml HGF (HGF 10), 50 ng/ml HGF (HGF 50), 100 ng/ml HGF (HGF 100), 10 ng/ml SCF (SCF 10), 50 ng/ml SCF (SCF 50), 100 ng/ml SCF (SCF 100), 10 ng/ml IGF2 (IGF2 10), 50 ng/ml IGF2 (IGF2 50), and 100 ng/ml IGF2 (IGF2 100) compared to the control. **c** Relative mRNA expression levels of stemness markers, *Nanog* and *OCT4*, per treatment group. **e** Western blot results and corresponding relative expression level of Runx2 protein per treatment group after 7 days of osteogenic induction. *P < 0.05, **P < 0.01, ***P < 0.001

We attempted to identify these key factors, as well as their effective concentrations, that are beneficial in maintaining the stemness of hBMSCs by culturing hBMSCs in DMEM supplemented with HGF, SCF, and IGF2. The hBMSCs were treated with different concentrations of HGF, SCF, and IGF2 for 3 days. Subsequently, the cell proliferation rate and expression levels of the stemness markers were measured. Furthermore, to determine the effects of HGF, SCF, and IGF2 on the osteogenic differentiation potential of hBMSCs, the 3 days of treatment was followed by 7 days of osteogenic induction. Results of the CFSE assay demonstrated that hBMSCs treated with 50 ng/ml HGF, 100 ng/ml HGF, 10 ng/ml SCF, and 10 ng/ml IGF2 had significantly increased cell proliferation, of which 100 ng/ml HGF had the strongest effect (Fig. 4a). In addition, the stemness markers, *Nanog* and *OCT4*, were highly upregulated in hBMSCs treated with 100 ng/ml HGF and 10 ng/ml SCF (Fig. 4b). Analysis of the osteogenic differentiation potential revealed that 100 ng/ml HGF and 10 ng/ml SCF greatly enhanced the expression levels of the osteogenic-related proteins, Runx2 (Fig. 4c). In total, these results demonstrate that 100 ng/ml HGF and 10 ng/ml SCF can effectively maintain the stemness of hBMSCs, while those treated with IGF2 only had a slight promoting effect or no effect. Therefore, 100 ng/ml HGF and 10 ng/ml SCF were finally determined for the following experiments, and the effects of the combination of the two factors were also observed.

### HGF and SCF delayed senescence and enhanced osteogenic differentiation potential of hBMSCs during long-term expansion

**Fig. 5:**
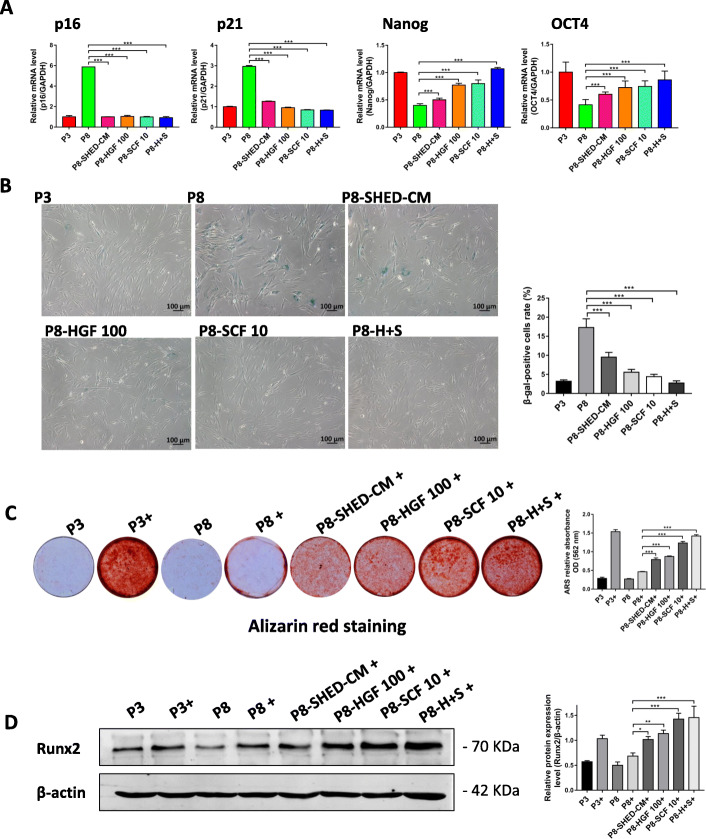
HGF and SCF delayed senescence and enhanced osteogenic differentiation potential of hBMSCs during long-term expansion. **a** Relative mRNA expression levels of *p16*, *p21*, *Nanog*, and *OCT4* in passage 3 (P3) and passage 8 (P8) hBMSCs cultured in SHED-CM (P8-SHED-CM) and treated with 100 ng/ml HGF (P8-HGF 100), 10 ng/ml SCF (P8-SCF 10), and the combination of 100 ng/ml HGF and 10 ng/ml SCF (P8-H+S). **b** β-Galactosidase stained hBMSCs and their corresponding β-gal-positive cell rates (%) per treatment group. **c** hBMSCs stained with alizarin red and the corresponding ARS relative absorbance OD of hBMSCs at 562 nm per treatment group. Plus sign indicates with osteogenic induction. **d** Western blot results and corresponding relative expression level of Runx2 protein per treatment group. *P < 0.05, **P < 0.01, ***P < 0.001

To determine the molecular mechanism underlying the cytokine-mediated maintenance of stemness, long-term expansion of hBMSCs from P3 to P8 in DMEM supplemented with HGF, SCF, or the combination of these two cytokines were performed. RT-qPCR results revealed that *p16* and *p21* expression levels significantly decreased while *Nanog* and *OCT4* expression levels increased in P8 hBMSCs treated with 100 ng/ml HGF or 10 ng/ml SCF (Fig. 5a). β-gal staining results confirmed that 100 ng/ml HGF or 10 ng/ml SCF prevented the senescence of late passage cells, and interestingly, the most significant effect was showed in hBMSCs treated with the combination of 100 ng/ml HGF and 10 ng/ml SCF (Fig. 5b). In addition, we determined the effects of HGF and SCF on the osteogenic differentiation ability of hBMSCs through alizarin red staining and the expression of the osteogenic protein, Runx2 (Fig. 5c, d). Results showed that hBMSCs cultured in DMEM with 100 ng/ml HGF or 10 ng/ml SCF after series expansion (P8) retained their excellent osteogenesis ability, which is equivalent to the osteogenesis ability of P3 hBMSCs (Fig. 5d). In total, these results demonstrate that 100 ng/ml HGF and 10 ng/ml SCF delayed senescence and enhanced osteogenic differentiation potential of hBMSCs during long-term expansion, and the most significant results was observed in hBMSCs treated with the combination of the two cytokines.

### HGF and SCF reduced ROS accumulation and preserved mitochondrial function of hBMSCs during long-term expansion

**Fig. 6:**
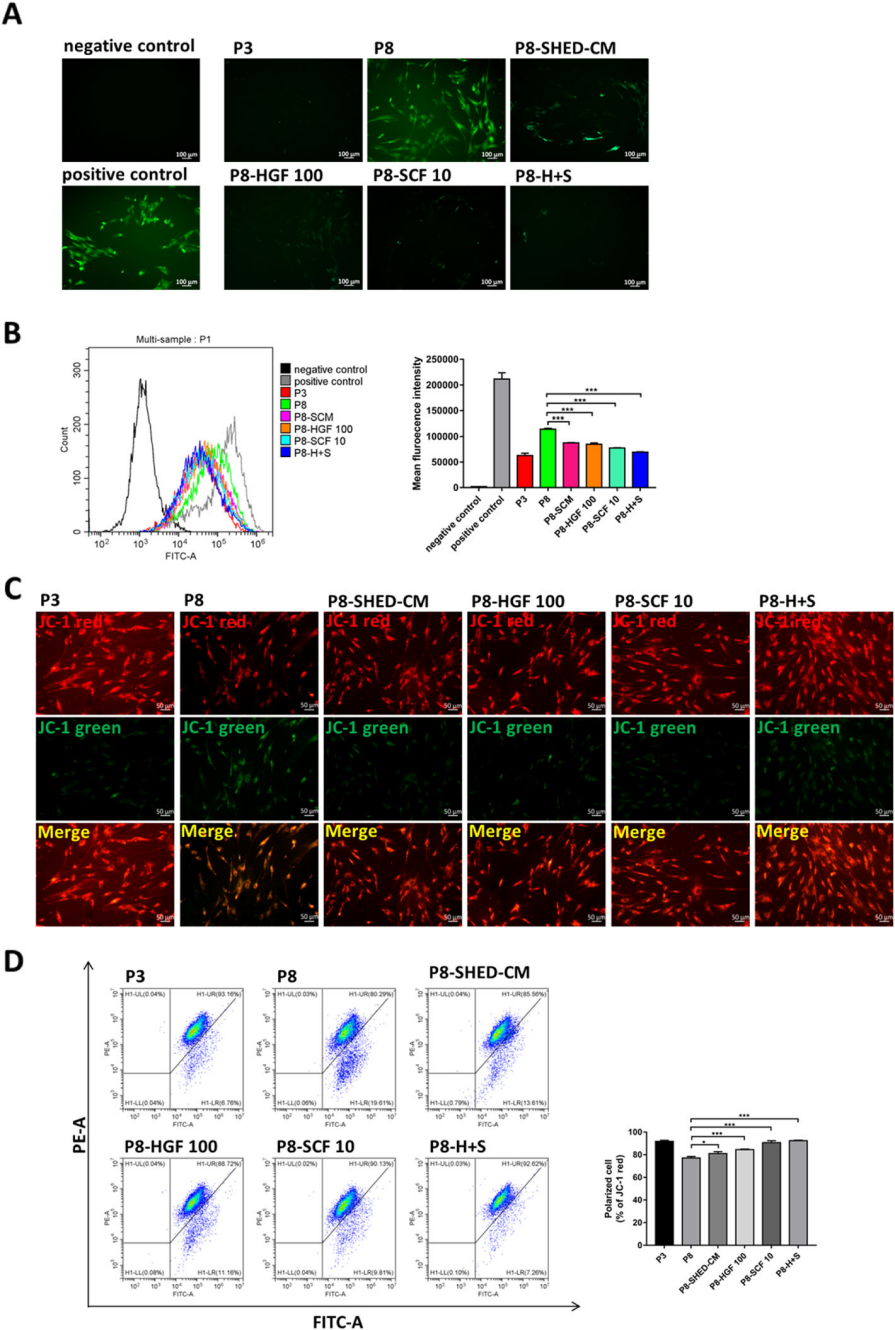
HGF and SCF reduced ROS accumulation and preserved mitochondrial function of hBMSCs during long-term expansion. **a** Representative images of ROS levels in negative control, positive control (stimulated with 1 mM H_2_O_2_ for 1 h), passage 3 (P3), and passage 8 (P8) hBMSCs cultured in SHED-CM (P8-SHED-CM) and treated with 100 ng/ml HGF (P8-HGF 100), 10 ng/ml SCF (P8-SCF 10), and the combination of 100 ng/ml HGF and 10 ng/ml SCF (P8-H+S). **b** Flow cytometry results of ROS levels and mean fluorescent intensity in hBMSCs per treatment group. **c** Representative images of mitochondrial membrane potential in hBMSCs per treatment group detected by JC-1 probe. The J-aggregates produced red fluorescence (JC-1 red); the monomer produced green fluorescence (JC-1 green). **d** Flow cytometry results of mitochondrial membrane potential and corresponding percentage (%) of polarized cell per treatment group detected by JC-1 probe. *P < 0.05, ***P < 0.001

The oxidative status of hBMSCs was also assessed by measuring the ROS production. Results showed that the ROS level of hBMSCs stimulated with 1 mM H_2_O_2_ for 1 h was significantly increased. hBMSCs in P8 had high ROS levels, while cells treated with 100 ng/ml HGF or 10 ng/ml SCF during long-term expansion had lower intracellular ROS levels. Especially the ROS levels in the group treated with the combination of the two cytokines significantly reduced the most (Fig. 6a, b). Long-term expansion in vitro can cause mitochondrial dysfunction, consequently promoting apoptosis. Here, the mitochondrial membrane potential was calculated using JC-1 mitochondrial membrane potential assay. Results showed that the mitochondrial membrane potential decreased in P8 hBMSCs compared with P3 hBMSCs. However, 100 ng/ml HGF- or 10 ng/ml SCF-treated hBMSCs exhibited a higher intensity of red fluorescence and a lower intensity of green fluorescence, indicating a higher level of mitochondrial membrane potential (Fig. 6c). Similar results were showed in flow cytometry detection that there were more polarized cells in cytokine-treated groups compared with P8 hBMSCs (Fig. 6d). These results suggest that 100 ng/ml HGF and 10 ng/ml SCF play an important role in preserving the mitochondrial functions of late passage cells.

### HGF and SCF preserved the mitochondrial function in hBMSCs via the PI3K/AKT, ERK1/2, and STAT3 signaling pathway

**Fig. 7:**
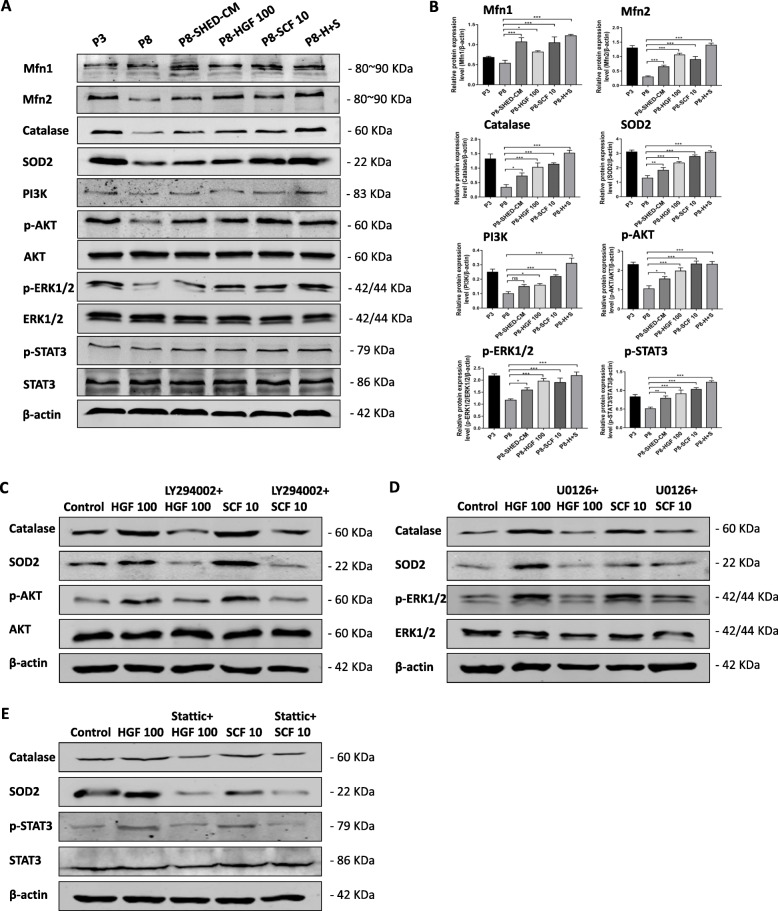
HGF and SCF preserved the mitochondrial function in hBMSCs via the PI3K/AKT, ERK1/2, and STAT3 signaling pathway. **a** Western blot results of the expression of mitochondrial-relative proteins (Mfn1, Mfn2, SOD2, and Catalase) and the PI3K/AKT, Erk1/2, and STAT3 signaling pathway-related proteins in passage 3 (P3) and passage 8 (P8) hBMSCs cultured in SHED-CM (P8-SHED-CM) and treated with 100 ng/ml (P8-HGF 100), 10 ng/ml SCF (P8-SCF 10), and the combination of 100 ng/ml HGF and 10 ng/ml SCF (P8-H+S). **b** Relative expression levels of the protein showed in the Western blot. ns, not significant. **c** Western blot results of the expression of mitochondrial oxidative stress-related proteins, Catalase and SOD2, and the PI3K/AKT signaling pathway-related proteins in P3 hBMSCs treated with 100 ng/ml HGF, 10 μM LY294002 plus 100 ng/ml HGF, 10 ng/ml SCF, and 10 μM LY294002 plus 10 ng/ml SCF. **d** Western blot results of the expression of mitochondrial oxidative stress-related proteins, Catalase and SOD2, and the PI3K/AKT signaling pathway-related proteins in P3 hBMSCs treated with 100 ng/ml HGF, 10 μM U0126 plus 100 ng/ml HGF, 10 ng/ml SCF, and 10 μM U0126 plus 10 ng/ml SCF. **e** Western blot results of the expression of mitochondrial oxidative stress-related proteins, Catalase and SOD2, and the PI3K/AKT signaling pathway-related proteins in P3 hBMSCs treated with 100 ng/ml HGF, 5 μM Stattic plus 100 ng/ml HGF, 10 ng/ml SCF, and 10 μM Stattic plus 10 ng/ml SCF. *P < 0.05, **P < 0.01, ***P < 0.001

To determine the mechanism of 100 ng/ml HGF or 10 ng/ml SCF responsible for maintaining mitochondrial function and stemness, the proteins associated with the PI3K/AKT, ERK1/2, and STAT3 signaling pathways were assessed using Western blot analysis. Results showed that late passage cells had significantly decreased expression of mitochondrial-related proteins. However, 100 ng/ml HGF or 10 ng/ml SCF treatments resulted in the increased expression of mitochondrial dynamics-related proteins, Mfn1 and Mfn2, and mitochondrial oxidative stress-related proteins, SOD2 and Catalase, which are the downstream molecules of the PI3K/AKT and ERK1/2 signaling pathways. Besides, Western blot analysis showed that 100 ng/ml HGF and 10 ng/ml SCF promoted the phosphorylation of AKT and ERK1/2. Therefore, we speculated that HGF and SCF preserved the mitochondrial function of hBMSCs via the PI3K/AKT and ERK1/2 pathways. In addition, the proteins related to the STAT3 pathway were activated and had significantly higher expression in the HGF- and SCF-treated groups than the P8 group (Fig. 7a). The PI3K/AKT and STAT3 pathways regulated the expression levels of the stemness markers, Nanog, and OCT4, and consequently the self-renewal ability of the stem cells, especially the STAT3 pathway, which is involved in regulating the cell cycle and apoptosis.

The PI3K/AKT, ERK1/2, and STAT3 inhibitors have been used in vitro experiment separately. hBMSCs in P3 were treated with DMEM that contained 10 μM LY294002 for 6 h before cytokine treatment to block PI3K/AKT pathway [27–29], 10 μM U0126 for 6 h to block ERK1/2 pathway [27, 30, 31], and 5 μM Stattic for 6 h to block STAT3 pathway [32–34]. Results showed that 100 ng/ml HGF and 10 ng/ml SCF promoted the phosphorylation of AKT, ERK1/2, and STAT3 compared with control group. The phosphorylation levels of AKT, ERK1/2, and STAT3 were all decreased when cells were treated with corresponding inhibitors, meanwhile the expression of mitochondrial oxidative stress-related proteins Catalase and SOD2 in cells were decreased by treatment of LY294002, U0126, and Stattic (Fig. 7c–e). Taken together, these results further verify that HGF and SCF treatment upregulated the expression of mitochondrial-related proteins Catalase and SOD2 via the PI3K/AKT, ERK1/2, and STAT3 signaling pathways, thereby preserving the mitochondrial functions and preventing the senescence of hBMSCs during long-term expansion.

## Discussion

In general, it is necessary to expand MSCs in vitro and obtain a sufficient quantity for use in clinical treatments. However, this is often difficult due to cell senescence, which is typically characterized by cell cycle arrest in G0/G1 phase (Fig. 1b), increased β-galactosidase activity (Fig. 2a), and upregulated expression of genes involved in cell cycle regulation, such as *p16* and *p21* (Fig. 2b). In the present study, we demonstrated that the microenvironment provided by SHED significantly reduced the senescence of hBMSCs after serial expansion.

Cell senescence also seriously affects the proliferation and functional activities of MSCs. To date, bone-related diseases are the primary focus in the clinical application and research of MSCs for therapy. However, the main challenge is how to reconstruct vascularized bone with physiologic structure and function. Here, we found that compared with P3 hBMSCs, P8 hBMSCs had almost negligible osteogenesis potential in vitro (Fig. 3). To address this, we focused on improving the microenvironment of MSCs in vitro by culturing hBMSCs in SHED-CM and identifying the key factors influencing the state of MSCs.

The formation of new blood vessels is a critical process during bone reconstruction and plays a major role in creating a niche for both the bone-forming skeletal stem cells and blood-forming hematopoietic stem cells [35, 36]. Our study revealed that transplanted MSCs in an aging state had significantly impaired bone-promoting capacity and significantly decreased pro-angiogenic capacity after series expansion (Supplemental Data 3). Senescent MSCs cannot induce angiogenesis in the damaged area, leading to local oxygen and nutrient deficiencies and exacerbated inflammation, which further impairs the activity and function of MSCs. Ultimately, this results in significantly decreased transplantation efficacy [37–39]. We also confirmed that SHED-CM enhanced the osteogenesis ability and delayed the senescence of hBMSCs, thereby resulting in better angiogenesis. The expansion of hBMSCs without loss of function provides new insights in creating strategies for the transplantation of MSCs and subsequent use in clinical therapy.

In this study, we demonstrated that SHED-CM could provide a better microenvironment for hBMSCs and maintain their stemness during long-term expansion. We also identified several key factors and possible mechanisms responsible for maintaining the stemness of hBMSCs and, consequently, design new methods to optimize the long-term expansion of MSCs in vitro.

HGF and SCF have attracted much attention because of their prominent expression in SHED. We observed that the addition of these factors to the culture medium significantly delayed the senescence (Fig. 5b), enhanced the osteogenic potential (Fig. 5c, d), and significantly reduced the accumulation of ROS in aging MSCs after long-term expansion (Fig. 6a, b). Excessive ROS in cells can activate stress-related signaling pathways, mediate mitochondrial inflammatory responses, cause mitochondrial function damage, and induce apoptosis [40–42]. Therefore, we further investigated the effects of HGF and SCF on the regulation of mitochondrial membrane potential (Fig. 6c, d), which is an important mitochondrial function that reflects the apoptotic state of cells. HGF and SCF enhanced the membrane potential of MSCs after series amplification and increased the production of Catalase and SOD2 that can catalyze ROS. Simultaneously, HGF and SCF upregulated the expression of mitochondrial dynamics-related proteins, specifically the fusion proteins Mfn1 and Mfn2, and increased the cellular oxidative phosphorylation and ATP synthesis [43, 44], which indicates that the mitochondrial activity of MSCs was effectively enhanced [45–47].

We also confirmed that HGF and SCF increased the expression of mitochondrial-associated proteins, Catalase and SOD2, via activation of the PI3K/AKT and ERK1/2 signaling pathways, which have anti-apoptotic roles and regulate the apoptosis-related proteins, Bcl and Bax [48]. HGF and SCF modulated the osteogenic differentiation of MSCs through the ERK1/2 signaling pathway [49, 50], effectively promoting the timely removal of excessive ROS in cells, reducing mitochondrial damage, and delaying cellular senescence. In addition, HGF and SCF can promote the self-renewal capacity of MSCs by regulating the expression of the cell cycle-related protein, p21, and the stemness markers, Nanog and OCT4, through the STAT3 pathway [51, 52]. Collectively, the findings of this study demonstrated that HGF and SCF have a potential application in studies focused on delaying cellular senescence of MSCs.

In this study, we have only confirmed that HGF and SCF have the effect of delaying senescence of MSCs after long-term expansion. However, there are still many other factors and molecular mechanisms waiting to be discovered. Furthermore, there are many structural and functional imbalances occur during the process of cellular senescence. We can also find the mechanism of HGF and SCF in maintaining stemness from other aspects, such as cell autophagy, DNA damage, and so on. Maybe we can find a new way to solve the problem of MSCs senescence from these areas.

## Conclusion

Both HGF and SCF are key factors in maintaining the stemness of hBMSCs by preserving mitochondrial function through the expression of proteins associated with the PI3K/AKT, ERK1/2, and STAT3 signaling pathways. This study provides new insights into the anti-senescence capability of HGF and SCF, as well as new evidence for their potential application in optimizing the long-term culture of MSCs.

## Supplementary information

**Additional file 1: Supplemental Data 1.** Characterization of SHED.

**Additional file 2: Supplemental Data 2.** Identification of key factors using RT-qPCR. **Table 2.** Primer sequences used in reverse transcription quantitative PCR (RT-qPCR).

**Additional file 3: Supplemental Data 3.** hBMSCs cultured in SHED-CM had enhanced pro-angiogenic capacity during long-term expansion.

## Data Availability

The datasets generated and/or analyzed during the current study are available from the corresponding author on reasonable request.

## Abbreviations

MSCs: Mesenchymal stem cells
SHED: Stem cells from human exfoliated deciduous teeth
hBMSCs: Human bone marrow mesenchymal stem cells
P3: Passage 3
P8: Passage 8
SHED-CM: Conditioned medium from SHED
RT-qPCR: Reverse transcription quantitative PCR
ALP: Alkaline phosphatase
HGF: Hepatocyte growth factor
SCF: Stem cell factor
cGVHD: Chronic graft versus host diseases
ECM: Extracellular matrix
IGF2: Insulin-like growth factor 2
hBMSCs-CM: Conditioned medium from hBMSCs
CFU: Colony-forming unit
PFA: Paraformaldehyde
PI: Propidium iodide
β-gal: β-Galactosidase
SDS-PAGE: Sodium dodecyl sulfate polyacrylamide gel electrophoresis
NC: Nitrocellulose
Mfn1: Mitofusin 1
Mfn2: Mitofusin 2
ARS: Alizarin red staining
ANOVA: One-way analysis of variance
