# Supplementary material for: Hepatocyte growth factor (HGF) and stem cell factor (SCF) maintained the stemness of human bone marrow mesenchymal stem cells (hBMSCs) during long-term expansion by preserving mitochondrial function via the PI3K/AKT, ERK1/2, and STAT3 signaling pathways
Source: Stem Cell Res Ther. 2020 Jul 31;11:329. doi: 10.1186/s13287-020-01830-4 (PMC7393921; doi:10.1186/s13287-020-01830-4)
Supplement: Supplementary file 1 — Additional file 1: Supplemental Data 1. Characterization of SHED. [file 13287_2020_1830_MOESM1_ESM.doc]

**Supplemental Data 1**

**Characterization of SHED**

SHED were seeded at a low density, and clearly formed colonies at day 14 (Supplemental Data 1A). The flow cytometry showed that SHED positively expressed CD44 (99.7%), CD90 (99.5%), and CD105 (97.9%) but negatively expressed CD34 (0.14%), CD45 (0.09%) and HLA-DR (0.169%) (Supplemental Data 1B). Tissue origin of SHED was identified by immunofluorescence. The results showed that SHED positively expressed Vimentin (a mesenchymal marker) (Supplemental Data 1C left) and negatively expressed Cytokeratin 18 (an epithelial marker) (Supplemental Data 1C right). To identify the multi-potency, we performed osteogenic and adipogenic differentiation assays. After incubation in osteogenic differentiation medium, cells could differentiate into osteoblasts as evidenced by Alizarin Red staining (Supplemental Data 1D left). After incubation in adipogenic differentiation medium, cells demonstrated adipocyte features as stained with Oil Red O (Supplemental Data 1D right).


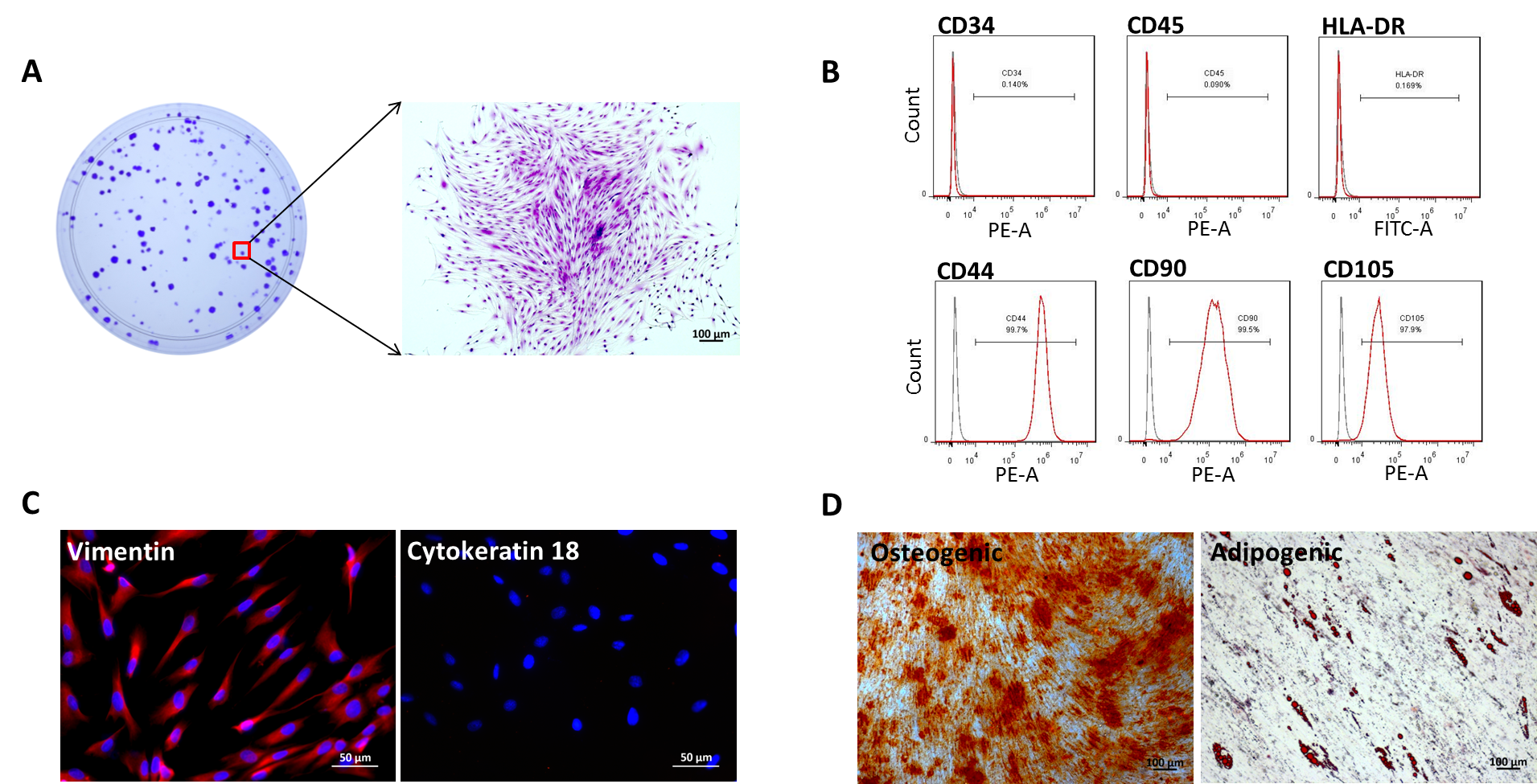


**Supplemental Data 1 Characterization of SHED.**

(A) Colony formation assay. (B) Surface markers identification. (C) Tissue origin identification. (D) Multiple differentiation ability demonstrated by Alizarin red (left) or Oil red O (right) staining.
