# Supplementary material for: Hepatocyte growth factor (HGF) and stem cell factor (SCF) maintained the stemness of human bone marrow mesenchymal stem cells (hBMSCs) during long-term expansion by preserving mitochondrial function via the PI3K/AKT, ERK1/2, and STAT3 signaling pathways
Source: Stem Cell Res Ther. 2020 Jul 31;11:329. doi: 10.1186/s13287-020-01830-4 (PMC7393921; doi:10.1186/s13287-020-01830-4)
Supplement: Supplementary file 2 — Additional file 2: Supplemental Data 2. Identification of key factors using RT-qPCR. Table 2. Primer sequences used in reverse transcription quantitative PCR (RT-qPCR). [file 13287_2020_1830_MOESM2_ESM.doc]

**Supplemental Data 2**


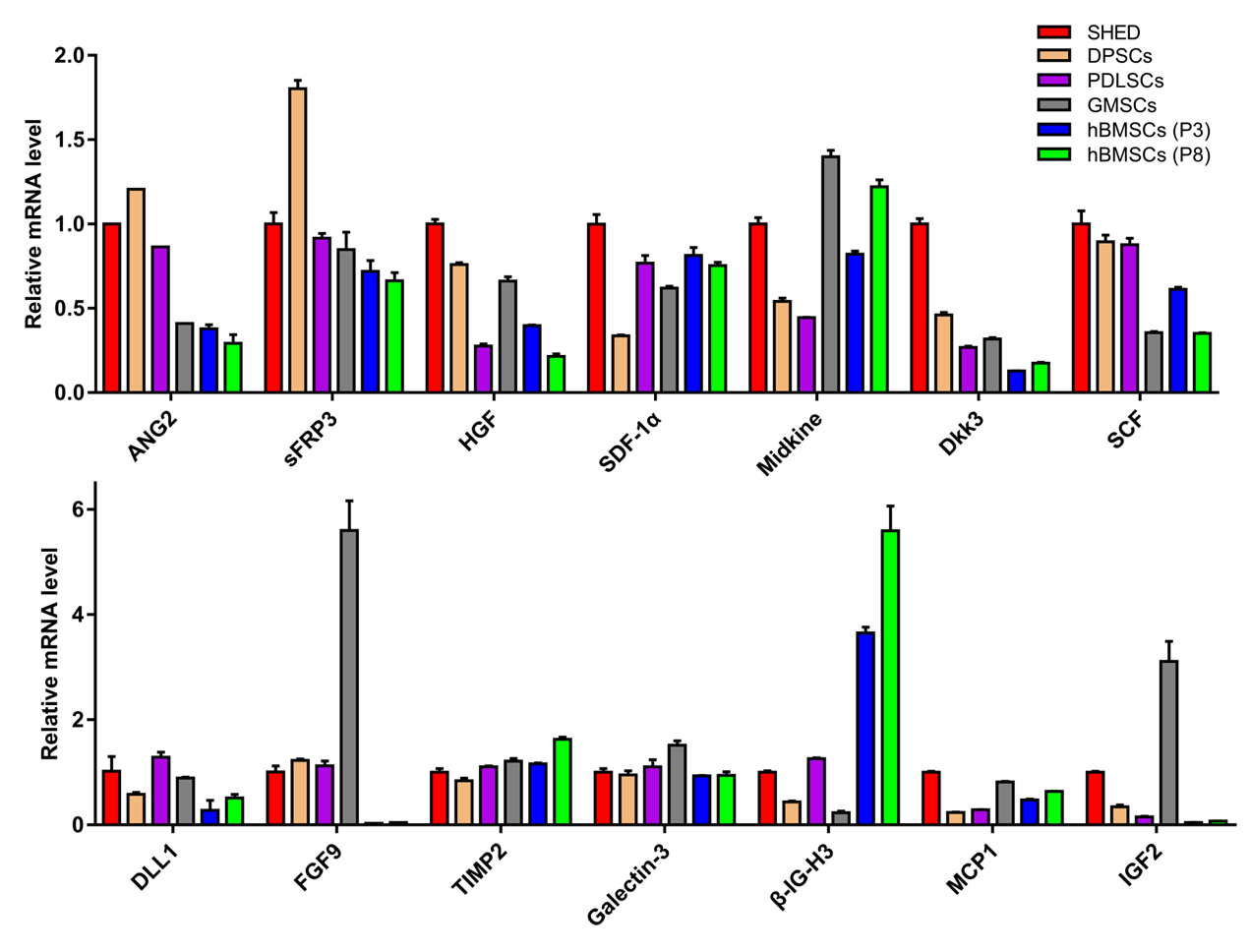


**Supplemental Data 2 Identification of key factors using RT-qPCR.**

The mRNA expression levels of cytokines in SHED, human dental pulp stem cells (DPSCs), periodontal ligament stem cells (PDLSCs), gingival mesenchymal stem cells (GMSCs) and hBMSCs in P3 and P8. ANG2: angiopoietin-2, sFRP3: secreted frizzled related protein 3, HGF: hepatocyte growth factor, SDF-1α: stromal cell-derived factor 1α, Dkk3: dickkopf-related protein 3, SCF: stem cell factor, DLL1: delta like canonical Notch ligand 1, FGF9: fibroblast growth factor 9, TIMP2: tissue inhibitor of metalloproteinase 2, β-IG-H3: TGF-beta inducible gene-h3, MCP1: monocyte chemoattractant protein-1, IGF2: insulin-like growth factor 2.

**Table 2** Primer sequences used in reverse transcription quantitative PCR (RT-qPCR).

| Gene | Sequence |
| --- | --- |
| ANG2 | Forward: 5’-AACTTTCGGAAGAGCATGGAC-3’  Reverse: 5’-CGAGTCATCGTATTCGAGCGG-3’ |
| sFRP3 | Forward: 5’-TGGAACATGACTAAGATGCCCA-3’  Reverse: 5’-ACACAGACTTACAGGGCTTGAT-3’ |
| HGF | Forward: 5’-GCTATCGGGGTAAAGACCTACA-3’  Reverse: 5’-CGTAGCGTACCTCTGGATTGC-3’ |
| SDF-1α | Forward: 5’-ATTCTCAACACTCCAAACTGTGC-3’  Reverse: 5’-ACTTTAGCTTCGGGTCAATGC-3’ |
| Midkine | Forward: 5’-CGCGGTCGCCAAAAAGAAAG-3’  Reverse: 5’-TACTTGCAGTCGGCTCCAAAC-3’ |
| Dkk3 | Forward: 5’-AGGACACGCAGCACAAATTG-3’  Reverse: 5’-CCAGTCTGGTTGTTGGTTATCTT-3’ |
| SCF | Forward: 5’-AATCCTCTCGTCAAAACTGAAGG-3’  Reverse: 5’-CCATCTCGCTTATCCAACAATGA-3’ |
| DLL1 | Forward: 5’-GATTCTCCTGATGACCTCGCA-3’  Reverse: 5’-TCCGTAGTAGTGTTCGTCACA-3’ |
| FGF9 | Forward: 5’-ATGGCTCCCTTAGGTGAAGTT-3’  Reverse: 5’-CCCAGGTGGTCACTTAACAAAAC-3’ |
| TIMP2 | Forward: 5’-AAGCGGTCAGTGAGAAGGAAG-3’  Reverse: 5’-GGGGCCGTGTAGATAAACTCTAT-3’ |
| Galectin-3 | Forward: 5’-ATGGCAGACAATTTTTCGCTCC-3’  Reverse: 5’-GCCTGTCCAGGATAAGCCC-3’ |
| β-IG-H3 | Forward: 5’-CTTCGCCCCTAGCAACGAG-3’  Reverse: 5’-TGAGGGTCATGCCGTGTTTC-3’ |
| MCP1 | Forward: 5’-CAGCCAGATGCAATCAATGCC-3’  Reverse: 5’-TGGAATCCTGAACCCACTTCT-3’ |
| IGF2 | Forward: 5’-GTGGCATCGTTGAGGAGTG-3’  Reverse: 5’-CACGTCCCTCTCGGACTTG-3’ |
| GAPDH | Forward: 5’-GGAGCGAGATCCCTCCAAAAT-3’  Reverse: 5’-GGCTGTTGTCATACTTCTCATGG-3’ |
