# Supplementary material for: Hepatocyte growth factor (HGF) and stem cell factor (SCF) maintained the stemness of human bone marrow mesenchymal stem cells (hBMSCs) during long-term expansion by preserving mitochondrial function via the PI3K/AKT, ERK1/2, and STAT3 signaling pathways
Source: Stem Cell Res Ther. 2020 Jul 31;11:329. doi: 10.1186/s13287-020-01830-4 (PMC7393921; doi:10.1186/s13287-020-01830-4)
Supplement: Supplementary file 3 — Additional file 3: Supplemental Data 3. hBMSCs cultured in SHED-CM had enhanced pro-angiogenic capacity during long-term expansion. [file 13287_2020_1830_MOESM3_ESM.docx]

**Supplemental Data 3**

**Methods**

**Tube formation assay *in vitro***

The supernatants of hBMSCs in each group (P3, P8, P8-SHED-CM and P8-hBMSCs-CM) were collected after 3 days cultured in DMEM. Tube formation assay were performed as below to evaluate the pro-angiogenic capacity of hBMSCs. Briefly, a 48-well culture plate was prepared with 100 μl matrigel matrix mixture (mixed with serum-free DMEM at a ratio of 1:1) (BD Biosciences, USA) per well in advance. Then, HUVECs were resuspended by the supernatants of hBMSCs, and seeded into plates at 6 × 10^4^ cells per well. After 4 h, 8 h and 24 h incubation at 37 °C, 5% CO_2_, tube formation in each group was detected under microscopy. The results of tube length, tube area, and the number of branch points were analyzed by ImageJ software.

**Results**

**hBMSCs cultured in** **SHED-CM had enhanced** **pro-angiogenic capacity during long-term expansion**

Tube formation assay was performed to assess the pro-angiogenic capacity of hBMSCs *in vitro*, which is an essential property of MSCs. Results showed that HUVECs migrate and begin to form small branches at 4 h. However, many tubes began to disconnect by 8 h in P8 and P8-hBMSCs-CM group. At 24 h, maximum tube formation was reached in P3 and P8-SHED-CM group, while there were no closed tubular structures remained in P8 and P8-hBMSCs-CM group. Overall, these results indicate that hBMSCs cultured in SHED-CM after long-term expansion also retained potential clinical applications in promoting neovascularization *in vivo*.


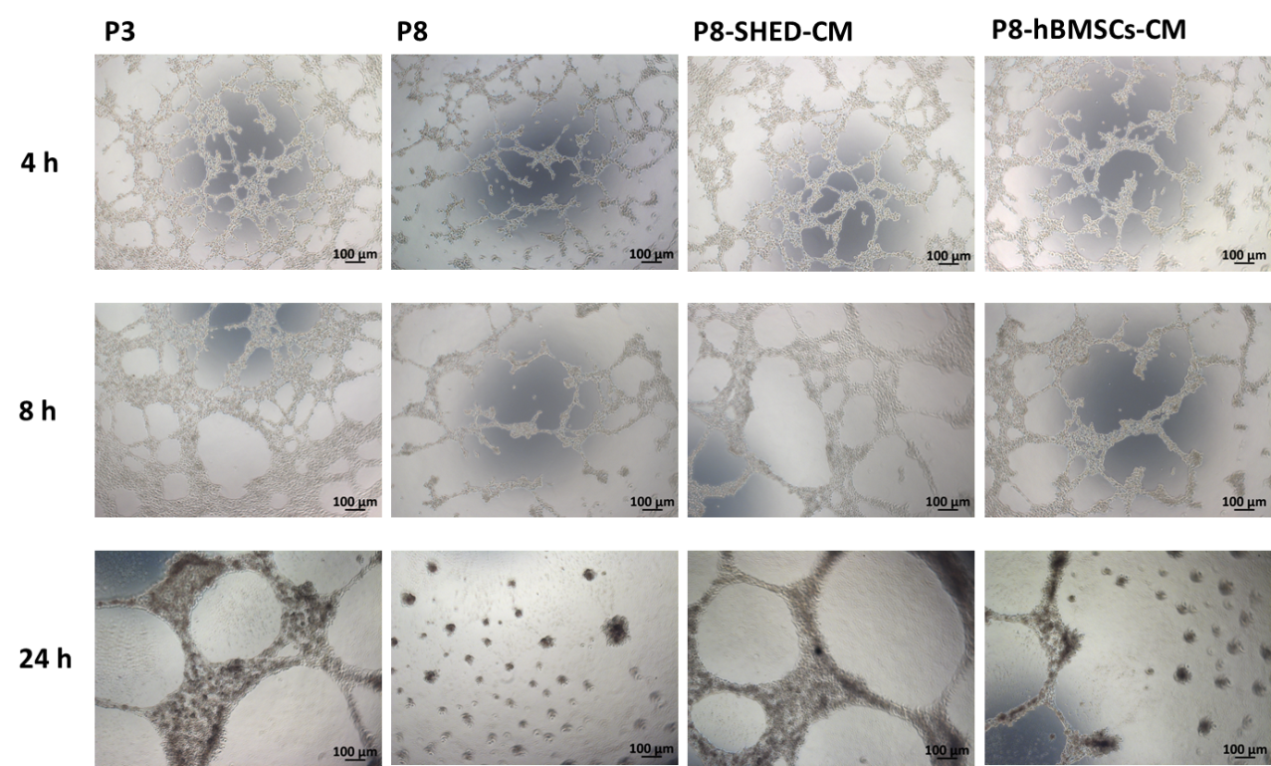


**Supplemental Data 3 hBMSCs cultured in SHED-CM had enhanced pro-angiogenic capacity during long-term expansion.**

The pro-angiogenic capacity of passage 3 (P3) and passage 8 (P8) hBMSCs cultured in DMEM, SHED-CM (P8-SHED-CM) and hBMSCs-CM (P8-hBMSCs-CM). Representative images of the tube formation assay per group when cells incubated for 4 h, 8 h and 24 h.
